# The Paradox Effect of Calcification in Carotid Atherosclerosis: Microcalcification Is Correlated with Plaque Instability

**DOI:** 10.3390/ijms22010395

**Published:** 2021-01-01

**Authors:** Manuela Montanaro, Manuel Scimeca, Lucia Anemona, Francesca Servadei, Erica Giacobbi, Rita Bonfiglio, Elena Bonanno, Nicoletta Urbano, Arnaldo Ippoliti, Giuseppe Santeusanio, Orazio Schillaci, Alessandro Mauriello

**Affiliations:** 1 Department of Experimental Medicine, University “Tor Vergata”, Via Montpellier 1, 00133 Rome, Italy; manuela.montanaro@uniroma2.it (M.M.); manuel.scimeca@uniroma2.it (M.S.); anemona@uniroma2.it (L.A.); francesca.servadei@ptvonline.it (F.S.); erica.giacobbi@ptvonline.it (E.G.); rita.bonfiglio@uniroma2.it (R.B.); elena.bonanno@uniroma2.it (E.B.); santeusa@uniroma2.it (G.S.); 2 Saint Camillus International University of Health Sciences, Via di Sant’Alessandro 8, 00131 Rome, Italy; 3 Fondazione Umberto Veronesi (FUV), Piazza Velasca 5, 20122 Milano, Italy; 4 Nuclear Medicine Unit, Department of Oncohaematology, Policlinico “Tor Vergata”, viale oxford 81, 00133 Rome, Italy; n.urbano@virgilio.it; 5 Vascular Surgery, Department of Biomedicine and Prevention, Policlinico “Tor Vergata”, viale oxford 81, 00133 Rome, Italy; ippoliti@med.uniroma2.it; 6 Department of Biomedicine and Prevention, University of Rome “Tor Vergata”, Via Montpellier 1, 00133 Rome, Italy; orazio.schillaci@uniroma2.it; 7 IRCCS Neuromed, 86077 Pozzilli, Italy

**Keywords:** atherosclerosis, plaque instability, carotid plaque, microcalcification, macrocalcification, macrophage polarization, inflammatory biomarkers

## Abstract

Background: this study aims to investigate the possible association among the histopathologic features of carotid plaque instability, the presence of micro- or macrocalcifications, the expression of in situ inflammatory biomarkers, and the occurrence of the major risk factors in this process in a large series of carotid plaques. Methods: a total of 687 carotid plaques from symptomatic and asymptomatic patients were collected. Histological evaluation was performed to classify the calcium deposits in micro or macrocalcifications according to their morphological features (location and size). Immunohistochemistry was performed to study the expression of the main inflammatory biomarkers. Results: results here reported demonstrated that calcifications are very frequent in carotid plaques, with a significant difference between the presence of micro- and macrocalcifications. Specifically, microcalcifications were significantly associated to high inflamed unstable plaques. Paradoxically, macrocalcifications seem to stabilize the plaque and are associated to a M2 macrophage polarization instead. Discussion: the characterization of mechanisms involved in the formation of carotid calcifications can lay the foundation for developing new strategies for the management of patients affected by carotid atherosclerosis. Data of this study could provide key elements for an exhaustive evaluation of carotid plaque calcifications allowing to establish the risk of associated clinical events.

## 1. Introduction

Vascular calcification is often associating with cardiovascular events and atherosclerosis [1,2,3,4]. In the past, it has been considered only a passive, degenerative process occurring at the end stage of atherosclerotic plaque formation in old patients. Recently, many clinic-pathological studies contributed to consider calcification as an active process correlated with the evolutive status of the atheromatous plaque [2,5,6,7]. From molecular point of view, some studies suggested that mineralization processes occurred in coronaries were similar to those involved in bone formation [8,9,10,11,12]. Nevertheless, molecular mechanisms implicated in the formation of plaque calcifications, as well as a better comprehension of the trigger events related to the calcium deposition, need further investigations. 

Among the most recognized processes involved in calcium apposition in atheromatous plaque there are death of smooth muscle cells (SMCs) and inflammatory cells, agglomeration of calcium-filled extracellular matrix vesicles, and decrease of mineralization inhibitors [8,9]. However, none of these mechanisms seems to be related to a specific morphological aspect of atheromatic plaques [13,14,15,16,17]. In addition, many studies suggest that the formation of plaque calcifications can be linked to inflammation-dependent mechanisms characterized by the activity of pro-inflammatory cells and specific macrophage polarization (M1 or M2) [2,8,18].

What is clear is that the progression of atheromatous plaques can follow different evolutions: in most patients vessels undergo a progressive narrowing of the lumen, which can lead to a chronic ischemia, even though, in absence of other conditions involved in tissue oxygenation reduction and/or increase tissue oxygen demand, no symptoms are developed. In a lower percentage of patients, the plaque undergoes a rapid evolution going towards vulnerability, rupture, and thrombosis, with the onset of acute symptoms (myocardial infarction, stroke and TIA which typically occur in plaques with mild or moderate stenosis) [19,20,21]. These two different types of evolution are related to two different categories of plaque: stable and unstable plaques, respectively [22,23]. 

Analyzing some of the putative mechanisms involved in the evolution of a vulnerable/unstable plaque, recent investigations tried to establish if size and location of calcific plaque, together with the process of calcification, may be crucial elements in the evolution of plaque instability [2,5,24,25]. In a previous study we demonstrated that elemental composition of calcification could influence the stability of atheromatous lesions [7,9]. In addition, the total artery calcium score, evaluated by cardiac computed tomography, represented a well-established marker of coronary plaque burden and is associated with a high risk of adverse cardiovascular outcomes [1,3]. Although coronary calcification is a marker of atherosclerosis, its effect on plaque instability seems to be less evident. As previously demonstrated by different studies, coronary calcification identifies the vulnerable patient rather than the vulnerable plaque. Indeed, the size of calcification in coronaries may be related to the plaque instability rather than the presence/absence of calcifications [6]. Specifically, Atsushi et al. showed that microcalcifications are frequently associated to coronary instable plaques [2]. Conversely, the presence of macrocalcifications seems to not affect the stability of coronaries, often characterizing the healing response to atherosclerotic inflammation. Nevertheless, only few studies have been performed about the possible role of micro- and macrocalcifications in the stability of carotid plaques [9,24,25,26]. 

Investigate carotid districts may be fundamental, since they present a larger vascular caliber as compared to coronaries, which already has been widely studied; moreover, to date, it is not yet clear whether calcifications interfere with the stability of the carotid plaques or represents only a passive phenomenon without any clinical significance. Also, the possible role of different risk factors on different plaque calcification processes still remains to be clarified.

Starting from these considerations, this study aims to investigate the possible association between the histopathologic features of carotid plaque instability and the presence of micro- or macrocalcifications in a large series of carotid plaques. Moreover, we have tried to verify the possible putative association among the presence of micro- or macrocalcifications the expression of in situ inflammatory biomarkers and the possible role of major risk factors in this process. 

## 2. Results

### 2.1. Clinical Data

The clinical characteristics of patients are reported in Table 1. 

The mean age of 687 patients at time of surgical carotid endarterectomy (CEA) was 68.8 ± 6.9 years. Of those, 476 (69.3%) were male and 211 (30.7%); 311 (45.3%) patients were symptomatic (affected by ipsilateral major stroke or transient ischemic attack), while 376 (54.7%) were asymptomatics who underwent CEA for high grade carotid stenosis. 

All patients included in the study had at least one risk factor. The hypertension was the risk factor most frequently observed (in 483 patients, 70.3%). Continuous treatment with aspirin (100 md/die) was administered to all patients in the post-operative and follow-up periods.

### 2.2. Calcification and Risk Factors

The presence of calcification in carotid plaque was not correlated to the presence of specific risk factors, as demonstrated by both uni- and multivariate analysis (see Table 2).

Only a significant correlation with the gender was observed: a higher incidence of calcifications were observed in female patients as compared to men. Indeed, calcified plaques were observed in 130 of the 211 (61.5%) female patients underwent CEA and in 239 of the 476 (50.2%) male patients (*p* = 0.05).

Similarly, neither microcalcifications nor macrocalcifications showed significant correlations with the main evaluated risk factors (Table 3). Since more than 90% of analyzed cases were over 60 years old, only a slight increase of plaque calcifications was observed in association with the age.

### 2.3. Histological Findings

In 348 out of 687 patients (50.7%) unstable plaques rich in inflammatory cells were found, consisting in 117 thrombotic plaques (all from symptomatic patients) associated with rupture of a thin and inflamed fibrous cap, 84 thin-cap fibro-atheroma TCFA (63 from symptomatic and 21 from asymptomatic patients) and 126 plaques with an organizing acute thrombus (all symptomatics) characterized by a network of large, thin-walled vascular channels and a variable number of macrophagic cells loaded with hemosiderin within the area of an acute thrombus. In 21 cases, plaques were characterized by a thin fibrous cap over calcified nodules which protruding into the lumen. In 5 of 21 cases, a discontinuity of the fibrous cap was observed with an overlying non-occlusive luminal thrombus. The remaining 339 carotids (49.3% of cases) showed a stable plaque characterized by a variable lipid-necrotic core containing extracellular lipid, cholesterol crystals, and necrotic debris covered by a thick fibrous cap with few inflammatory cells. 

Uni- and multivariate statistical analysis demonstrated that calcifications were significantly more present in stable plaques, as compared to unstable ones. Specifically, the presence of calcifications was observed in 203 out of 339 stable plaques (59.9%) and in 166 out of 348 unstable plaques (47.7%) (*p* = 0.001). In this last group, calcifications were observed in the 44.8% of thrombotic plaques and in 42.9% of vulnerable ones (excluding calcific nodules). 

Stable fibrocalcific plaques showed a thick, fibrous cap overlying extensive accumulations of calcium in the intima close to the media, associated to a small lipid-laden necrotic core. In this type of plaque, unlike the calcified nodules, calcium had a non-nodular aspect and it not protruded into the lumen as occur for eccentric nodules.

### 2.4. Plaques with Microcalcifications vs. Those with Macrocalcifications

**Figure 1:**
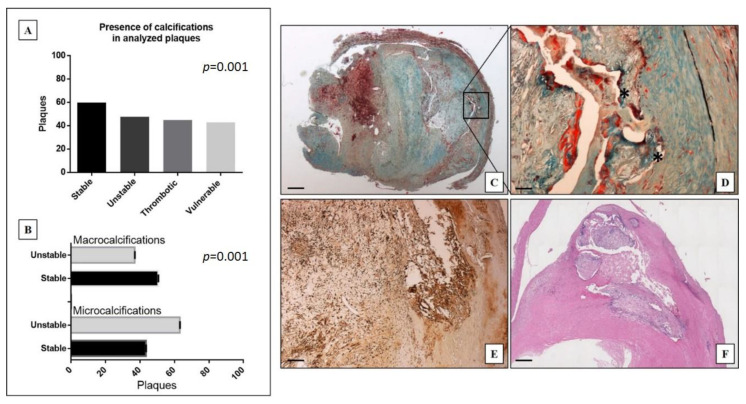
Distribution and morphological analysis of calcifications in carotid plaques. (**A**) Graph shows the presence of calcifications in stable, unstable, thrombotic, and vulnerable plaques. (**B**) Graph displays the presence of micro or macrocalcification in stable and unstable plaques. (**C**) Movat staining shows the presence of microcalcifications in an unstable atheromatous plaque with acute thrombosis in organization. Square indicate the area enlarged in panel D (scale bar represents 500 µm). (**D**) A magnification of microcalcification reported in panel C (scale bar represents 50 µm). (**E**) Immunohistochemical staining shows numerous CD163 positive M2 macrophages in the thrombotic atheromatous plaque reported in panel C (scale bar represents 100 µm). (**F**) Hematoxylin and Eosin staining of a stable carotid plaque with macrocalcification, constituted mainly by fibrous tissue (scale bar represents 500 µm).

Data here reported showed a significant distribution of the presence calcification in stable, unstable, thrombotic and vulnerable plaques (*p* = 0.001) (Figure 1A). As shown in the Figure 1B microcalcifications (n = 152) were significantly more frequent in unstable plaques (56.6%) than in stable ones (63.1%) (*p* = 0.001). On the contrary macrocalcifications were observed in 137 stable plaques (63.1%) and in 80 unstable ones (36.9%) (Figure 1B). As compared to macrocalcifications, microcalcifications were usually placed close to the tunica media (Figure 1C,D). In particular, microcalcifications showed a distance from the vascular lumen of 2.5 ± 0.1 mm compared to 1.8 ± 0.2 mm of the macrocalcifications (*p* = 0.001). Lastly, microcalcifications were present in plaques with a wider atheroma (relative area of atheroma was 24.9 ± 2.0% in plaques with micro and 16.9 ± 1.1% in those with macrocalcifications, *p* = 0.001).

The analysis of the subpopulations of inflammatory cells carried out on the TMA has highlighted the presence of a greater number of CD163 and CD86 positive cells in plaques with macro, as compared to those with microcalcifications (*p* = 0.01 and *p* = 0.02, respectively) (Table 4) (Figure 1E,F). In addition, a significant increase in the TGF-beta expression was observed in plaques with macrocalcifications as compared to those with microcalcifications (*p* = 0.04) (Table 4).

## 3. Discussion

The study of the mechanisms involved in the formation of ectopic calcifications, as well as the characterization of the prognostic value of these calcium deposits, represent an intriguing challenge for biomedical research. Indeed, the presence of ectopic calcifications is considered a clinical sign of tissue degeneration in numerous human diseases such as atherosclerosis, breast cancer, prostate cancer, and neurological disorders [9,11,12,27]. 

In this scenario, atheromatic calcifications are used by clinicians to establish the risk of cardiovascular events, mainly in coronary plaques. Specifically, a scoring system based on cardiac computed tomography analysis is commonly use in the clinical practice as a well-established marker of coronary plaque burden [3,5,28,29]. No specific scoring systems have been developed to evaluate the risk associated to the presence of calcium deposits in carotid plaques since the lack of solid and inconvertible data. Also, the cellular and molecular mechanisms involved in the formation of carotid plaque calcifications, as well as the possible role of micro- and macrocalcifications in plaque stability, have not been fully identified. 

Therefore, this study aims to investigate in a large series of carotid plaques the possible association between the histopathologic features of carotid plaque instability and the presence of micro- or macrocalcifications. Moreover, we have tried to verify the possible putative association among the presence of micro- or macrocalcifications the expression of in situ inflammatory biomarkers and the possible role of major risk factors in this process. 

Results of our study performed in a large series of cases demonstrated that calcifications are very frequent in carotid plaques, with a significant difference between the presence of micro and macrocalcifications. Specifically, microcalcifications were significantly associated to high inflamed unstable plaques. Paradoxically, macrocalcifications seem to give stability to the plaque. It is worth noting that an increase in the macrophage infiltrate was observed in carotid plaque with macrocalcifications, especially for what concerns the presence of CD163 positive cells (M2 macrophages).

Data here showed highlighted a possible different mechanism in the formation of macro- and microcalcifications in carotid plaques. Historically, the presence of macrocalcifications was considered the last event of the accumulations of a large number of microcalcifications [30]. The evidence that the presence of micro- and macrocalcifications is related to a different macrophages’ polarization could provide a scientific rationale capable to explain both the genesis of these calcium deposits and their different association with the carotid plaque stability. As already established for coronary plaques [31], our results suggest that carotid plaque calcifications develop by the inflammation-dependent mechanisms involved in progression and regression of atherosclerosis. The presence of high number of M1 macrophages in carotid instable plaques with microcalcifications could trigger the calcium deposition in the context of the atheroma’s necrotic core by inducing vesicle-mediated mineralization process related to both macrophages and vascular smooth muscle cells (VSMCs) apoptosis [32]. Thus, the formation of carotid microcalcifications could be considered an inflammatory degenerative plaque process rather than an active phenomenon similar to bone mineralization. On the other hand, it is known that the late phases of plaque development can be characterized by a regression process in which M2 macrophages play a crucial role in the healing response to inflammation [2]. When this occurs, plaques generally exhibit the peculiar characteristics of a stable plaque. Besides reducing inflammation into the atheroma, M2 macrophages can induce the differentiation of several cell types into osteoblast-like cells capable to product large amount of calcium crystals and thus inducing the formation of macrocalcifications [33]. This phenomenon has been described in several human diseases including the coronary plaques [34]. In these plaques, the M2 macrophages could induce the differentiation of VSMCs into vascular osteoblast-like cells but also differentiate themselves into cells capable to product calcifications. In this scenario, it is important to note that we reported a positive association between the TGF-beta expression and the presence of macrocalcifications, thus supporting the hypothesis of an active involvement of M2 macrophages in the formations of these calcium deposits. Indeed, TGF-beta is considered a key element of bone metabolisms since it is able to stimulate the synthesis of bone matrix protein and the deposition of calcium crystals [35,36]. Also, it has been established that M2 macrophages express high-level of TGF-beta [37]. The expression of this molecule into the carotid atheroma by M2 macrophages could sustain the massive production of calcifications inducing the osteoblastic differentiation of VSMCs. Therefore, the activity of M2 macrophages into carotid atheroma can stabilize the carotid plaques by reducing the inflammation and inducing the formation of large calcium deposits (macrocalcifications). However, some studies suggested that the presence of carotid macrocalcifications, despite stabilizing the atheromatic lesion, can induce the destabilization of areas placed downstream of plaque calcification by altering the shear stress [4]. 

From clinical point of view, the two types of carotid plaque calcifications have distinct implications. Macrocalcification leads to plaque stability, while microcalcification is more likely to be associated with plaque rupture. In addition, carotid calcifications have pleiotropic properties as pro-inflammatory microcalcification and anti-inflammatory macrocalcification. Conversely, the formation of micro or macrocalcification is not specifically related to the main cardiovascular risk factors. 

The large case selection investigated in this study allows us to speculate about the possible use of carotid calcifications, micro or macro, as clinical markers of carotid plaque burden. Moreover, a better characterization of the molecular mechanisms involved in the formation of carotid calcification may provide data for developing new instrumental analysis capable to early detect unstable plaques. 

Molecular imaging analysis such as positron emission tomography (PET), single photon emission computed tomography (SPECT), or CT could reach these goals by using molecules capable to bind calcifications or highlight M2 macrophages into the carotid atheroma [38]. In particular, recent studies proposed the use of ^18^-NaF PET/CT to detect and quantify microcalcification in early-stage of atheromatic plaque formation. It is demonstrated that the uptake of NaF correlates with cardiovascular risk factors and appears to be a good measure of the body’s atherosclerotic burden, potentially suited also for assessment of anti-atherosclerotic therapy [39,40]. Also, Barrut et al. [41] used CT angiography (CTA) images, to localized calcified carotid plaques and more important to study the topographic anatomy of these calcifications thus identified possible morphological aspects capable to predict the risk of plaque burden.

As concern possible limitations, in this study were only collected plaques from patients submitted to CEA during a given time frame of their diseases natural and therefore the here reported case selection could not be representative of the whole population affected by carotid atherosclerosis. In addition, sampling at 5-mm intervals along the length of each plaque may potentially miss additional plaque features occurring only between the points of sectioning.

## 4. Material and Methods

### 4.1. Cases Selection

A total of 687 carotid plaques from symptomatic (major stroke or TIA) and asymptomatic patients submitted to surgical carotid endarterectomy (CEA) at the University of Rome Tor Vergata from 2010 to date were studied. All asymptomatic patients showed a carotid stenosis >60%, assessed by echography or, in rare cases, by bilateral CT angiography.

Informed consent was obtained from each patient. The study protocol was approved by the IRBs of our Institution (reference no. 129.18, 26 July 2018).

### 4.2. Histology

Only intact calcified carotid plaques from patients which undergone CEA and for whom complete clinical and laboratory assessment of major cardiovascular risk factors were included in the study and histologically analyzed. The sampling collection and analysis methods have been previously reported [19]. Briefly, samples were fixed immediately upon removal in 10% buffered formalin for 24 h. All plaques were cut transversely every 5 mm, embedded in paraffin and stained with hematoxylin-eosin. 

### 4.3. Histological Classification

Plaques were divided, according to the modified American Heart Association atherosclerosis classification [23,28], into stable and unstable. Unstable plaques were constituted by (a) thrombotic plaques associated with rupture or erosion of the cap; (b) healed plaque with a thrombus in organization; (c) vulnerable plaque or thin-cap fibro-atheroma (TCFA) characterized by a fibrous cap less than 165 µm thick heavily infiltrated by macrophages, CD68 positive (>25 per high magnification field), without plaque rupture. The other plaques were classified as stable.

In each plaque, the presence and type of calcification were evaluated. Calcification was divided in micro and macrocalcification. Calcium deposits of ≤0.5 mm were defined as microcalcifications. On the contrary calcifications >0.5 mm were considered as macrocalcifications. Moreover, in each plaque was evaluated the distance of calcium from the vessel lumen, the lipidic-necrotic core area, the presence of intraplaque hemorrhage and the grade of plaque inflammation.

Histopathologic examination was performed by two different pathologists (AM, FS) blinded to the clinical data. Interobserver reliability was >98%.

### 4.4. TMA Construction 

In order to perform the detailed evaluation of 22 immunohistochemical markers of the inflammatory process a tissue microarray (TMA) was constructed as previously reported [42]. Briefly, defined areas, which include the cap and the shoulder of a small series of plaque with micro and macrocalcifications were selected. Plaque were randomly selected from our cases and were matched by age and sex with the above series. The master block (receiving paraffin block) with all 73 spot samples was built by the Galileo TMA CK3500 (Brugherio, Milan, Italy). 

### 4.5. Immunohistochemistry 

Immunohistochemical analysis was performed in all plaques in order to characterize the presence of monocyte/macrophages cells using CD68 and CD3 antibodies. Moreover, immunohistochemistry was also performed on 14 TMA serial sections to characterize the in-situ expression of the main inflammation markers as reported in Table 5.

Immunohistochemical reactions were evaluated by counting the number of cells/mm^2^. Briefly, antigen retrieval was performed on 3-μm-thick paraffin sections using citrate pH 6.0 or EDTA citrate pH 7.8 buffers for 30 min at 98 °C. Sections were then incubated for 1 hour at room temperature with primary antibodies. Reactions were revealed by HRP-DAB Detection Kit (UCS Diagnostic, Italy). To assess the background of immuno-staining a negative control for each reaction was performed by incubating sections with only secondary antibodies (HRP) and detection system (DAB). Reactions have been set-up by using specific control tissues as indicated in datasheets.

### 4.6. Risk Factors Definition

Clinical records were reviewed for all cases to determine risk factor profile. The risk factors were defined according to the following criteria: (a) hypertension: patients with systolic BP ≥ 140 mmHg and/or a diastolic BP ≥ 90 mmHg without antihypertensive treatment or taking antihypertensive treatment at the time of carotid endarterectomy; (b) diabetes mellitus: patients with fasting blood glucose > 126 mg/dL and/or following oral treatment or insulin therapy; (c) patients with tobacco dependence were categorized as smokers if they smoked more than 10 cigarettes/day. Former smokers who had stopped smoking for >5 years were considered as non-smokers; (d) hypercholesterolemia: patients with total cholesterol level > 200 mg/dL (>5.18 mmol/L); (e) patients with low HDL-C: <40 mg/dL in men or <50 mg/dL in women; (f) hypertriglyceridemia: patients with serum triglycerides levels ≥ 150 mg/dL (>1.70 mmol/L).

### 4.7. Statistical Analysis 

Data were analyzed using SPSS version 16.0 (SPSS Inc, Chicago, Ill) software. Continuous variables were expressed as the mean ± SD or ± SE. The Shapiro–Wilk test was used to statistically assess the normal distribution of the data. Comparisons between continuous variables were performed using the independent Student’s *t*-test or the Wilcoxon rank sum test. Categorical data were analyzed using the chi square test or the Fisher exact test. 

Multivariate analysis using stepwise logistic regression (using the ‘enter’ method for variable selection) was utilized to identify independent risk factors which significantly correlate with the presence of different type of calcification. The following variables were included: age, gender, hypertension, diabetes, smoking habit, hypercholesterolemia, low HDL, hypertriglyceridemia, and statin therapy. Multivariate analysis was performed in 2 models: (1) absence of calcification vs. presence of calcification; (2) micro vs. macrocalcification.

A two-tailed *p*-value < 0.05 was considered statistically significant.

## 5. Conclusions

Carotid atherosclerosis is related to the occurrence of very disabling clinical events which frequently compromise the patient’s quality of life. The characterization of possible mechanisms involved in the formation of carotid calcifications, as well as the identification of possible biomarkers associated to plaque instability, can lay the foundation for developing new strategies for the management of patients affected by carotid atherosclerosis. Data of this study, obtained on a large case selection, could provide key elements for a correct and exhaustive evaluation of carotid plaque calcifications allowing to establish the relative risk of plaque burden. 

## Figures and Tables

**Table 1:** Baseline characteristics of patients.

	*n* (%) or Mean (SD)
Total Age	*n* = 687 68.8 (6.9)
Gender Male Female	476 (69.3%) 211 (30.7%)
Cerebrovascular disease Symptomatic patients Ipsilateral major stroke TIA Asymptomatic patients	311 (45.3%) 117 (17.0%) 194 (28.2%) 376 (54.7%)
Risk factors Hypertension Diabetes Smoking habit Hypercholesterolemia Hypertriglyceridemia Low-HDL	483 (70.3%) 197 (28.7%) 459 (66.8%) 457 (66.5%) 339 (49.3%) 289 (42.1%)
Drugs Statins Diuretics	412 (60.0) 275 (40.0)
Associated vascular disease Previous myocardial infarction Peripheral arterial diseas Aortic aneurysm	137 (19.9) 185 (26.6) 34 (4.9)
Histological type of carotid plaque Stable plaques Unstable plaques Thrombotic plaque With a thrombus in organization TCFA Calcified nodule	339 (49.3%) 348 (50.7%) 117 (17.0) 126 (18.3%) 84 (12.2%) 21 (3.1%)

**Table 2:** Correlation between the presence of calcifications and risk factors

	Absence of Calcification *n* = 318	Presence of Calcification *n* = 369	Univariate Analysis *p*	Multivariate Analysis *p*
Age, mean (SD)	68.3 (6.9)	69.4 (6.7)	0.25	0.07
Gender Male Female	237 (74.5%) 81 (25.5%)	239 (64.8%) 130 (35.2%)	0.11	0.05
Hypertension	211 (66.3%)	261 (70.7%)	0.51	0.51
Diabetes	94 (29.6%)	98 (26.6%)	0.71	0.84
Smoking habit	201 (63.2%)	246 (66.7%)	0.31	0.82
Hypercholesterolemia	209 (65.7%)	234 (63.4%)	0.86	0.10
Hypertriglyceridemia	138 (43.4%)	190 (51.5%)	0.29	0.97
Low HDL Statins	118 (37.1%) 189 (59.4%)	166 (45.0%) 223 (60.4%)	0.19 0.67	0.13 0.12

**Table 3:** Correlation between the type of calcifications and risk factors

	Microcalcification *n*= 152	Macrocalcification *n*= 217	Univariate Analysis *p*	Multivariate Analysis *p*
Age, mean (SD)	69.7 (7.5)	69.1 (6.4)	0.59	0.11
Gender Male Female	107 (7046%) 45 (29.6%)	132 (60.8%) 85 (39.2%)	0.07	0.35
Hypertension	105 (69.1%)	156 (71.9%)	0.69	0.84
Diabetes	42 (27.6%)	56 (25.8%)	0.81	0.24
Smoking habit	103 (67.8%)	143 (65.9%)	0.80	0.25
Hypercholesterolemia	99 (65.1%)	135 (62.1%)	0.77	0.96
Hypertriglyceridemia	77 (51.7%)	113 (52.1%)	0.91	0.60
Low HDL Statins	73 (48.0%) 93 (61.2%)	93 (42.9%) 130 (59.9%)	0.63 0.74	0.89 0.35

**Table 4:** Correlation between type of calcification and histological markers of inflammation and mineralization

	Microcalcification	Macrocalcification	*p*-Value
CD86	2.28 + 0.82	8.22 + 2.28	0.02
CD163	11.60 + 3.91	33.56 + 7.55	0.01
CD44	6.17 + 3.12	3.40 + 0.95	0.41
CD53	53.62 + 13.72	52.70 + 7.57	0.95
CD57	4.61 + 2.82	4.13 + 1.26	0.88
CD197	3.04 + 1.80	0.95 + 0.19	0.27
IL2	30.39 + 8.54	47.80 + 7.84	0.14
IL6	48.15 + 11.54	65.13 + 6.70	0.22
IL10	8.62 + 7.38	5.23 + 1.59	0.66
IL17	19.88 + 7.20	31.21 + 6.38	0.25
IL23	12.2 + 7.4	29.6 + 4.8	0.06
TGF-β	10.09 + 5.50	24.37 + 4.00	0.04

**Table 5:** Immunohistochemical antibodies for characterization of inflammation and mineralization markers expression

Antibody	Characteristics	Antigen Retrieval and Dilution
CD3	Rabbit Monoclonal, clone 2GV6; Ventana, Tucson, AZ, USA	pH 7.8, 1:100
CD68	Rabbit Monoclonal, clone KP-1; Ventana, Tucson, AZ, USA	pH 7.8, 1:100
CD86	Rabbit Monoclonal, clone C86/2160R (ab234401); Abcam, Cambridge, UK	pH 7.8, 1:100
CD163	Rabbit Monoclonal, clone EPR19518 (ab182422); Abcam, Cambridge, UK	pH 7.8, 1:100
CD44	Rabbit Monoclonal, clone EPR18668 (ab189524); Abcam, Cambridge, UK	pH 7.8, 1:100
CD53	Rabbit Monoclonal, clone EPR4342(2), (ab134094); Abcam, Cambridge, UK	pH 7.8, 1:100
CD57	Mouse Monoclonal, clone NK-1 (ab233872); Abcam, Cambridge, UK	pH 7.8, 1:100
CD197	Rabbit Monoclonal, clone EPR23192-57 (ab253187), Abcam, Cambridge, UK	pH 7.8, 1:100
IL2	Rabbit Monoclonal, clone EPR6452 (ab205859); Abcam, Cambridge, UK	pH 7.8, 1:100
IL6	Mouse Monoclonal (ab9324); Abcam, Cambridge, UK	pH 7.8, 1:100
IL10	Rabbit Polyclonal (ab34843); Abcam, Cambridge, UK	pH 7.8, 1:100
IL17	Rabbit Polyclonal (ab9565); Abcam, Cambridge, UK	pH 7.8, 1:100
IL23	Rabbit Polyclonal (ab45420); Abcam, Cambridge, UK	pH 7.8, 1:100
TGF-β	Rabbit Monoclonal, clone EPR21143 (ab215715); Abcam, Cambridge, UK	pH 7.8, 1:100

## Data Availability

The data presented in this study are available on request from the corresponding author. The data are not publicly available due to privacy concerns.
